# Recent advances in understanding and treatment of Parkinson’s disease

**DOI:** 10.12703/b/9-6

**Published:** 2020-11-10

**Authors:** Arjun Tarakad, Joseph Jankovic

**Affiliations:** 1 Parkinson’s Disease Center and Movement Disorder Clinic, Department of Neurology, Baylor College of Medicine, 7200 Cambridge, Suite 9A, Houston, TX 77030-4202, USA

**Keywords:** Parkinson’s Disease, emerging treatment, pathogenesis

## Abstract

Though primarily a sporadic condition, Parkinson’s disease is increasingly recognized to be a multifactorial disease with a strong genetic component. At a cellular level, disruptions of protein trafficking and recycling, particularly by misfolding, accumulation, and aggregation of α-synuclein, mitochondrial dysfunction, oxidative stress, and other etiopathogenic mechanisms, have been found to result in the death of vulnerable neuronal populations and appear to drive the neurodegeneration underlying Parkinson’s disease. The improved understanding of these mechanisms has led to the development of novel pathogenesis-targeted and potentially disease-modifying therapeutic approaches in Parkinson’s disease. Until these treatments are fully developed and approved, clinicians must rely on therapies designed to improve quality of life of patients by treating various motor and non-motor symptoms of the disease.

## Introduction

Parkinson’s disease (PD) is a neurodegenerative disorder classically characterized by a combination of rest tremor, slowness (bradykinesia), increased muscle tone (rigidity), and impairment of gait and balance. In addition to exhibiting these cardinal motor symptoms, patients with PD can exhibit a wide array of motor and non-motor symptoms, including changes in posture and other skeletal deformities (for example, “striatal hands”), loss of sense of smell and taste, autonomic dysfunction, mood disturbances, cognitive impairment, sleep disturbances, and pain. Although the disorder has long been considered to be a primarily sporadic disorder (despite frequent family history of PD), the identification of numerous PD-related genes since the late 1990s has changed this perception and definition of PD^1^. Indeed, many now view PD as a syndrome with different pathogenic mechanisms producing characteristic or atypical symptoms. Genetic susceptibility coupled with environmental factors and aging seems to drive the development of PD^2^.

In line with this changing perception of PD to a syndrome, there has been increasing interest in identifying PD “subtypes” based on clinical phenotype, underlying genotype, and pathology^3^. Of course, the ultimate goals are to better understand that pathogenesis and develop disease-specific and progression biomarkers that will lead to individualized symptomatic and disease-modifying therapies^4^.

In this review, we briefly discuss the most common PD-related genes along with proposed mechanisms of etiopathogenesis based on affected pathways and the role of environmental exposure in the development of PD. We also highlight new findings related to the interaction between gut and brain. We then discuss recent advances in the development of potential disease-modifying therapies and more personalized treatment.

## Genetics in Parkinson’s disease

About 5 to 10% of patients with PD have a monogenic form of the disease following classic Mendelian inheritance patterns, and the remaining cases are felt to be sporadic, although over 100 susceptibility genes and risk-associated gene variants have been identified^5,6^. Many of these gene variants are linked to pathways involved in autophagy and lysosomal biology and other cellular mechanisms that impair clearance of rogue proteins, such as α-synuclein.

After the 1997 discovery of the gene that codes for α-synuclein (*SNCA*) and subsequent identification of α-synuclein as the major component in Lewy bodies (the hallmark pathologic finding in PD), basic and clinical research shifted focus on this protein as one of the important factors in understanding the pathogenesis of neurodegeneration in PD^2,7,8^. Oligomerization of α-synuclein as a result of mutant *SNCA* and accumulation of excess protein due to duplication or triplication of the *SNCA* gene have been postulated as mechanisms of PD in these rare patients with *SNCA* mutations or multiplications, although this concept of α-synuclein as a toxic protein has been extended to other forms of PD, including “idiopathic”^2^. As oligomeric α-synuclein continues to aggregate, it becomes insoluble and forms β sheet–rich amyloid aggregates that impair cell function^9^. This α-synuclein pathology spreads and propagates in a prion-like fashion. Indeed, prion-like spread has been postulated as a mechanism of progression in many neurodegenerative diseases besides PD (synucleinopathies and other proteinopathies)^10–12^.

Mutation in the *LRRK2* gene, encoding for leucine-rich repeat kinase 2 protein, first mapped in 2002^13^, is much more commonly implicated than *SNCA*, accounting for 4% of familial and 1% of sporadic cases of PD, but in certain populations may account for up to 10% of all “sporadic” PD and 42% of familial cases in Europe and North Africa, particularly in North African Berbers, Iberian populations, and Ashkenazi Jews (13% sporadic and 30% familial)^14,15^. It is worth noting that *LRRK2* mutations show a great degree of phenotypic and pathologic heterogeneity as well as variable penetrance, ranging from 20 to 100%. This variability is not readily explained by the specific mutations.

Several genes, when mutated, can affect mitochondrial function and result in a PD phenotype. The *Parkin* gene mutations are associated with younger-onset recessively inherited PD, accounting for 10 to 50% of cases of PD with onset before 50 years of age^16,17^. A notable pathologic feature includes the paucity of Lewy bodies as compared with idiopathic PD^18^. Mechanistically, the Parkin protein is involved in ubiquitination and protein recycling and plays an important role in mitochondrial homeostasis^19^. The *PINK1* gene encodes for phospatin and tensin (PTEN) homolog-induced kinase 1, a protein that localizes to mitochondria and associates with the ubiquitinating Parkin protein to regulate mitophagy^19,20^. The *DJ-1* gene encodes a peptidase that protects against oxidation, and mutations in the gene are associated with altered mitochondrial morphology^5,21^. Both *PINK1* and *DJ-1* mutations result in a recessively inherited young-onset PD clinically similar to the Parkin phenotype^1^. The growing understanding of the role of mitochondria, highly concentrated in striatal nerve terminals, has led to an evolving concept that PD represents an axonopathy with initial axonal arborization and mitochondrial dysfunction in the striatum with subsequent striatal-nigral degeneration^22–24^.

The *VPS35* gene encoding vacuolar sorting protein 35 is associated with an autosomal dominant form of PD showing reduced penetrance and late onset of disease^5,25,26^. VPS35 is a subunit of a protein complex known as the retromer, which is involved in recycling of membrane proteins through association with endosomes and facilitation of transport to the trans-Golgi network or plasma membrane. VPS35 dysfunction results in enhanced accumulation of insoluble α-synuclein and disruption in mitochondrial function and turnover^26^.

Mutations in *GBA*, the gene encoding glucocerebrosidase, have been associated with the recessively inherited lysosomal storage disorder Gaucher’s disease. This gene has been garnering increasing attention because of growing recognition of a high prevalence in certain PD populations; as much as 10 to 15% of sporadic cases and over 40% of familial cases in the Ashkenazi Jewish population carry the *GBA* gene mutation^27^. Several other genes have been implicated in lysosomal storage disorders and have been associated with risk of developing PD^28,29^.

Genome-wide association studies have expanded the number of loci associated with increased genetic risk of PD and have explained 11 to 15% of the heritable risk of PD^6,30^. It is likely that additional genes will be identified in the future and will provide further evidence of heritability of PD, but the challenge will be to explain how these genetic risks translate into clinical and pathological phenotypes^31^.

## Extrinsic factors in Parkinson’s disease

A causal role of an environmental factor in PD is often difficult to prove. This is because, as with other neurodegenerative disorders, inciting events in the disease process may predate clinical symptoms by years or even decades. Several environmental factors, including pesticide exposure, rural living, and heavy metal exposure, have been associated with an increased risk of developing PD^32^. Furthermore, consumption of dairy products, exposure to methamphetamine, and co-morbid medical conditions such as type 2 diabetes mellitus, autoimmune disorders, traumatic brain injury, or melanoma have been associated with increased risk of developing of PD^32,33^. In contrast, cigarette smoking, caffeine consumption, and green and black tea consumption appear to confer a reduced risk of PD development^32^. Beta blockers have been proposed to increase the risk of developing PD, whereas beta agonists have been suggested as protective, possibly through proposed up- and down-regulation of *SNCA* expression, respectively^34,35^, although some population-based studies have failed to show this correlation^36^. Low uric acid levels have also been associated with an increased risk of PD across many studies^37,38^, although recent biomarker studies have challenged this observation^39^.

## Gut–brain relationship

The gastrointestinal system has been known to be involved in PD, as evidenced by the frequent occurrence of constipation even during the prodromal phase, but its role in the pathogenesis of PD was not recognized until quite recently. One of the leading hypotheses behind the progression of PD is the transmission of synuclein pathology from the periphery through olfactory neurons and from the enteric nervous system via the vagus nerve, followed by centripetal spread to the substantia nigra; this is the so-called dual-hit hypothesis proposed by Braak and colleagues^40^. The finding of α-synuclein aggregates in the gastrointestinal tract supports the notion of spread of synuclein pathology from the gut via the vagus nerve to the caudal brainstem and then rostrally to the substantia nigra, diencephalon, and neocortex^24,40–42^. Further support for the involvement of the gut and the vagus nerve in the pathogenesis and progression of PD are studies that demonstrate a modest protective effect of vagotomy in the development of PD^43,44^. The make-up of gut microbiota has been shown to differ in PD patients relative to healthy controls and may even correlate with disease severity and symptoms^44–46^, raising the possibility that different gut microbiota contribute to the etiopathogenesis of the disease^47^.

Although α-synuclein has been at the center stage as a key protein implicated in the pathogenesis of PD, it is worth noting that its pivotal role and the hypothesis of Braak and colleagues have been challenged^48^. For example, it is important to note that the staging of Braak and colleagues is based on Lewy body pathology rather than neuronal loss and that up to a third of patients do not show Lewy body pathology in the enteric system. Finally, some have suggested that protein aggregation represents a compensatory response to cellular stress (epiphenomenon)^49^.

## Pathogenesis-targeted treatment strategies in Parkinson’s disease

One of the outcomes of improved understanding of pathogenic mechanisms underlying PD is the development of more targeted treatments toward these mechanisms^8^. For example, there has been a great deal of interest in antibodies targeting α-synuclein^50,51^, given its prevalence and presumed pathogenic role in a majority of cases of PD. Other potential disease-modifying treatments being investigated in PD include glucagon-like peptide-1 receptor agonists (currently used in treatment of diabetes), nilotinib (a chemotherapeutic agent) and its analogs, and numerous gut dysbiosis trials aiming to “normalize” gut microbiota of patients with PD (Table 1)^2,52–55^.

**Table 1.** Emerging pathogenesis-targeted treatments in Parkinson’s disease.

Category	Treatment agent(s)	ClinicalTrials.gov Identifiers	Trial phase	Proposed mechanism of action
Alpha-synuclein immunotherapy	PRX002 BIIB054 ABBV-0805	NCT03100149 NCT03318523 NCT03716570 NCT04127695	Phase 2 Phase 2 Phase 1 Phase 1	Passive immunization via infused alpha- synuclein antibodies
	PD01A PD03A	NCT01568099 NCT02618941 NCT02267434	Phase 1 Phase 1 Phase 1	Active immunization via administration of synthetic peptide sequences
Tyrosine kinase inhibitor	Nilotinib K0706	NCT03205488 NCT02954978 NCT03655236	Phase 2 Phase 2 Phase 2	Inhibition of Abelson tyrosine kinase (which inhibits Parkin)
Glucagon-like peptide 1 agonist	Exenatide Semaglutide Liraglutide	NCT04269642 NCT04305002 NCT04154072 NCT04232969 NCT03659682 NCT02953665	Phase 2 Phase 2 Phase 2 Phase 3 Phase 2 Phase 2	Acts on MAP kinase and PI3 kinase to decrease neuroinflammation
Gut dysbiosis	Fecal transplant	NCT03671785 NCT03876327 NCT03808389	Phase 1 Phase 2/3 --	Microbiota transfer via fecal transplantation from healthy donor
	Resistant maltodextrin	NCT03667404	Phase 2	Prebiotic treatment to regulate gut microbiome
Glucocerebrosidase-targeted therapy	Ambroxol	NCT02914366	Phase 2	Increase levels of beta- glucocerebrosidase to lower alpha- synuclein levels
	PR001A	NCT04127578	Phase 1/2a	*GBA* gene delivered to neurons via viral vector

Though not pathogenesis-targeted treatments *per se*, a number of new dopamine replacement strategies, including adeno-associated virus-mediated gene therapy to boost dopamine production in surviving neurons, exogenously induced dopaminergic neuron precursor cells, or even the conversion of astrocytes into neuronal cell populations, represent exciting new prospects for the field of PD therapeutics^56–59^. Also, cell-based therapies, including autologous-induced pluripotent stem cells, are being intensely investigated as potential therapies in PD^60^.

There is a growing body of evidence that PD is not a single clinical-pathological entity but a syndrome consisting of multiple disease states with different underlying mechanisms of neurodegeneration, hence requiring a specific (personalized) therapeutic approach^61^. Furthermore, even within PD, there may be subtypes, such as the tremor dominant subtype, postural instability and gait difficulty (PIGD) subtype, and other subtypes^3,62^. Beyond these subtypes, different genetic forms of PD also demonstrate unique or characteristic phenotypes, including age at onset, presence of dystonia and other movement disorders, development of complications to levodopa therapy, deep brain stimulation (DBS) responsiveness, and a degree of associated cognitive impairment^1^. Better understanding of underlying disease mechanisms as well as identification of different phenotypes by genetic analysis opens the door to incorporating subtype-specific, personalized treatments in PD^3,31,49^. Research into such experimental therapies, such as LRRK2 inhibitors and treatments targeting enhancement of glucocerebrosidase activity or facilitating substrate clearance in *GBA*-associated PD, has already reached planning and trial phases^28^.

One of many challenges in the development of disease-modifying therapies has been marked paucity of clinical, biochemical, or imaging markers that reliably track the progression of the disease^63^. The clinical gold standard for assessing severity of PD symptoms and signs, the UPDRS or the MDS-UPDRS, used in nearly all clinical trials as a primary outcome measure, has many limitations, including marked within-subject variability^64^. Technology has also started to play an increasing role in PD diagnosis and treatment with the hope of finding digital biomarkers that may allow earlier detection of PD or further discrimination among subtypes. However, difficulty in interpretation of gathered data and lack of standardization across device platforms are current barriers to widespread reliable usage^65,66^.

## Conclusions

Recent years have witnessed a dramatic increase in our understanding of genetics in PD and consequently an improved understanding of pathogenic and pathophysiologic mechanisms of the disease. These include discoveries about α-synuclein and its role as a “rogue protein”, reduced protein clearance, mitochondrial dysfunction, and oxidative stress. We are entering an exciting era during which the improved knowledge about cellular and molecular mechanisms underlying neurodegeneration in PD is translated into pathogenesis-specific, disease-modifying treatments in PD and a more personalized approach designed to slow or prevent progression of the disease and improve quality of life of patients with PD.
